# Intimate partner violence and its contribution to mental disorders in men and women in the post genocide Rwanda: findings from a population based study

**DOI:** 10.1186/s12888-014-0315-7

**Published:** 2014-11-18

**Authors:** Aline Umubyeyi, Ingrid Mogren, Joseph Ntaganira, Gunilla Krantz

**Affiliations:** Department of Epidemiology and Biostatistics, School of Public health, College of Medicine and Health Sciences, University of Rwanda, Kigali, Rwanda; Department of Public Health and Community Medicine, Sahlgrenska Academy at Gothenburg University, Gothenburg, Sweden; Department of Clinical Sciences, Obstetrics and Gynecology, Umea University, Umea, Sweden

**Keywords:** Intimate partner violence, Mental disorder, Men, Women, Genocide, Rwanda

## Abstract

**Background:**

In low income countries, mental disorders are a neglected health problem. Mental disorders are influenced by a number of factors in people’s everyday life of which intimate partner violence (IPV) commonly form an important part. The aim of this study was to investigate the prevalence of mental disorders in young men and women in Rwanda and their risk factors with main emphasis on IPV and its contribution to mental disorders, taking into account the genocide context.

**Methods:**

This population-based study included a representative sample of 917 men and women aged 20-35 years. The prevalence of mental disorders was investigated using of a diagnostic tool, the “MINI: Mini International Neuropsychiatric Interview”. Risk factor patterns were analysed with bi- and multivariate logistic regression. To find the proportion of mental disorders attributed to IPV, the population attributable fraction was computed.

**Results:**

The prevalence rates of current depression, suicide risk and PTSD were more than two times higher in women than in men while for generalized anxiety disorder, the prevalence was about the same. Physical, sexual and psychological intimate partner violence exposure was highly associated with all forms of mental disorders for women. For physical violence, after adjusting for socio-demographic factors and exposure to traumatic episodes during the Rwandan genocide, the risk of current depression for women was elevated four times. Even though few men reported partner violence exposure, physical violence in the past year was found to be a statistically significant risk factor for current depression and for generalized anxiety disorder. However, having an experience of traumatic episodes during the genocide contributed to the risk of most of mental disorders investigated for men.

**Conclusion:**

In Rwanda, IPV contributed considerably to mental disorders investigated. Thus, prevention of IPV should be considered as a public health priority, as its prevention would considerably reduce the prevalence of mental disorders.

## Background

Mental disorders constitute a threat to individuals, families and countries well-being; about 450 million people worldwide suffer from a mental disorder [1]. These kinds of diseases contribute to about 14% of the global burden of disease [1] and 30% of the total non-communicable disease burden all over the world [2]. Moreover, mental disorders are among the most neglected conditions worldwide [2], they strike young people in their productive and reproductive age, with devastating effects not only for the victim but for the entire family. However, it often goes unrecognized and untreated in most low and middle-income countries [3].

Studies are increasingly demonstrating that intimate partner violence (IPV) has a negative impact on women’s health [4,5]. At a global level, it is acknowledged that IPV directed towards women is associated with a number of health effects such as injuries, mental health problems, HIV, pain and cardio-vascular problems [5]. The latest World Health Organization (WHO) report on IPV and its health effects indicates that women who are physically or sexually abused by their partners are almost twice as likely to experience depression compared to unexposed women [4]. A review of studies in high income countries has shown a variation in IPV prevalence across countries and, irrespective of the difference in magnitude, IPV is associated with a range of mental disorders including depression, PTSD, anxiety, self-harm and sleep-disorders [5]. A study from Australia has found that IPV among 18-44 year old women accounted for 7.9% of the overall burden of disease [6].

The situation in low and middle income countries is comparable [7,8]. A study conducted in Ethiopia highlighted that physical violence, high spousal control of women by the partner and psychological violence were independently associated with depression after adjusting for socio-economic factors [9]. In a Vietnamese study of a similar design, women exposed to IPV were at a substantially higher risk of memory loss, pain or discomfort, sadness or depression and suicidal thoughts compared to those with no IPV experience in the past year [10].

Rwanda is a low-income country, located in central Africa, with a population of 10.5 million inhabitants and a population density among the highest in Africa (416 inhabitants/ km^2^) [11]. Similar to other low-and-middle-income countries, non-communicable diseases (NCD) are becoming more and more predominant [12,13]. Formerly considered as diseases of the wealthy, NCDs are today competing with infectious diseases in this population, indicating a country in an epidemiological transition phase.

The 2008 update for the Global Burden of Disease Study estimated that non-communicable conditions accounted for 17% of the disease burden in Rwanda in 2004, of which neuropsychiatric conditions contributed to approximately 4% [14] while the country was still spending only 1% of its health budget on mental health [13].

A study from Rwanda investigating the association between IPV and mental disorders, with married men and women, using the Self-Reporting Questionnaire (SRQ-20) to measure common mental disorders and the Revised Conflict Tactics Scale for violence items, indicates that physical violence exposure was associated with common mental disorders and suicidal ideation [15]. Only few studies exist on men’s exposure to IPV in Sub-Saharan Africa and there is hardly any study on associated health effects. In our earlier study on IPV prevalence, women are much more exposed to physical, sexual and psychological intimate partner violence than men during past year and earlier in life periods [16].

Furthermore, the Rwandan genocide in 1994, during which about 800.000 people were killed still negatively influences mental health of Rwandan population [17]. As years pass, it becomes more and more evident that there is a considerable degree of psychiatric morbidity as a consequence of serious violent acts inflicted during the Rwandan genocide [17,18]. A recent study investigating the trauma exposure related to the Rwandan genocide establish a clear picture of long-term imprints, where a good proportion of the study population was diagnosed with depressive and anxiety symptoms associated with the genocide 17 years later [19].

Prevalence studies on mental health in low income countries are limited while studies on IPV are on the increase; however limited attention has been paid to the association between the two in a population based study including both men and women in post conflict countries.

This study explored the prevalence of mental disorders in a population of young men and women in Rwanda and their risk factors with main emphasis on IPV and its contribution to mental disorders. However, as the Rwandan genocide still causes mental disorders in many Rwandans, the contribution of such exposure was also explored in both men and women.

This study forms part of a larger project on violence and other traumatic episodes, mental health and barriers to care among young men and women, *The Rwandan Violence, Mental Health and Barriers to Care project (RwVMHBC-project)*.

## Methods

### Study design, study population and sample size

A population-based cross-sectional study was conducted on a representative sample of young adults, aged 20 to 35 years, chosen because they represent a large group of the Rwandan population and further, are vulnerable to violence [20] and mental disorders [4]. The sample size was calculated based on the prevalence of depression (20%) in Rwanda [21], a desired level of absolute precision of 5% and an estimated design effect of 1.5. In all, 917 households were included. As only one interview was conducted in each household for ethical reasons, the final sample was comprised of 440 men and 477 women with two refusals to participate.

A multi-stage random sampling was used to find households for inclusion in the survey. The selection of villages (the smallest administrative unit in Rwanda), households and study participants in the eight districts of the Southern province of Rwanda was made in three phases. Out of the total number of 3512 existing villages, 35 villages were randomly selected using a random function. The number of households in each village was selected proportionate to the total number of households in each village and the person to be interviewed was randomly selected among eligible people in each household i.e. men and women aged between 20 and 35 years. The first participant in each village was selected from the closest household to the center of the village and a calculated sampling interval was applied to get the next household.

### Instruments

To assess the mental health status, four modules from the MINI International Neuropsychiatric interview (DSM-IV) version 5.0.0, were used to identify major depressive episodes in the past two weeks and in earlier periods of 2 week-duration or more, generalized anxiety disorder, suicide risk and post-traumatic stress disorder (PTSD). The MINI is designed as a brief structured interview for diagnosing the major psychiatric disorders according to DSM-IV criteria and International Classification of Diseases-10. Validation studies show that the MINI has similar validity and reliability properties as the WHO-CIDI (Composite International Diagnostic Interview for ICD-10) instrument [22]. The advantage with the MINI is that it contains less items, takes less time to administer and can be used by trained interviewers but does not necessarily require clinical staff. To measure physical, sexual and psychological violence exposure, we used the items from the Women’s Health and Life experiences questionnaire, developed by WHO [23] and used in several countries all over the world [24]. The questionnaires were translated in Kinyarwanda, one of the national languages in Rwanda.

### Data collection procedures

The data collection took place between December 2011 and January 2012. The School of Public Health, College of Medicine and Health Sciences, University of Rwanda, was the lead implementer of the survey. A pool of experienced interviewers composed of 13 clinical psychologists and two supervisors were recruited. Two-day training was carried out followed by one day for questionnaire piloting. The data entry was performed by four skilled personnel from the Rwanda School of Public Health under the supervision of a data entry manager.

### Measurements

#### Dependent variables

Five mental disorders were used as dependent variables: *major depressive episodes current (in the 2 weeks preceding the survey)*, *major depressive episodes past (earlier in life periods of two weeks or more)*, *generalized anxiety disorder (in the past six months)*, *suicide risk (anytime in life)* and *post-traumatic stress disorder current (in the past month*) (PTSD).

The major depressive episodes current, generalized anxiety disorder and PTSD sections of the MINI start with two screening questions corresponding to the main criteria of the disorder and end with a diagnostic conclusion indicating whether the criteria was met or not. For suicide risk, consisting of six questions related to symptoms, diagnosis was reached when one was met.

For each mental disorder, the diagnostic conclusion was dichotomized into ‘experience’ as the exposed category set against ‘no experience’ as the reference category.

#### Independent variables

Physical, sexual and psychological intimate partner violence, traumatic episodes during the genocide period and socio-demographic variables, were analysed as independent risk factors.

*Intimate partner violence* occurrence was measured by the three forms of violence, physical, sexual and psychological violence in the year preceding the survey. The past year prevalence was measured by assessing acts of physical (6 acts), sexual (3 acts) and psychological (4 acts) violence [16]. The composite measures for physical, sexual and psychological violence respectively were formed and dichotomised into any, as opposed to no violence experience. The ‘no violence’ category contained persons with no physical, sexual or psychological violence experience.

*Age* was described as a three category variable (20-24 years, 25-29 years, and 30-35 years), then grouped into 2 categories (20-29 years and 30-35 years) for further analyses. M*arital status* was divided into married/cohabiting, divorced/widowed and single, later dichotomized as married/cohabiting as the exposed. The *Educational level* was described as a three group variable (secondary school/university, complete primary/vocational training and incomplete primary); for the bivariate and multivariate analyses; education level was divided into incomplete primary contrasted with higher education. *Personal income per month* was categorized as a three category variable with more than 35,000Rwf (60USD), between 17,500-35,000Rwf (30-60 USD) and less than 17,500Rwf (30 USD) and further, grouped into earning 17,500Rwf or more per month as the reference category and less than 17,500Rwf per month constituted the exposure category. *Social support* was defined as having a friend or family member that will assist if you become ill, would share food, share housing, lend money, help you with guidance when problems arise and offer support when having personal problems. A composite variable of social support was constructed, grouped into good social support corresponding to, having ‘always’, ‘often’ or ‘sometimes’ the kind of support inquired about, as opposed to poor social support equivalent to the absence of a positive response to any of the support items.

The available *assets in the household* included a radio, a television set, a refrigerator, a bicycle, a motorcycle, a car, a mobile phone and a computer. The assets were merged and dichotomised into having at least one of the items (reference category) versus having none of the items (exposure category). This variable was used as proxy for socio-economic status and having none of the assets inquired about constituted the very poor.

Given the possibility that any existing mental disorder may partly be associated with exposure to any traumatic event during the Rwandan genocide [25], the association between IPV and mental health effects was adjusted for the traumatic episodes during the genocide period. The *Traumatic episodes during the genocide period* was a summary measure of items of traumatic episodes from the revised versions of Harvard Trauma Questionnaire [26] related to the genocide exposure, dichotomized into “any” as opposed to “no traumatic experience” [27].

### Statistical analysis

Risk factors were estimated with odds ratios (OR) and their 95% confidence interval (CI) in bi- and multivariate analyses to estimate the association between violence exposure and mental disorders. IBM SPSS Statistics version 20 was used for all descriptive, bivariate and multivariate statistical analyses.

In the multivariate analyses, the final model was created for each mental disorder with each form of violence as the main independent factor. The logistic regression analyses were adjusted for variables proving statistical significance with a majority of mental disorders in the bivariate analyses. For women, these were partner’s low education, lack of assets in the household and experience of traumatic episodes during the genocide. For men, the variables controlled for were lack of assets in the household and exposure to traumatic episodes during the genocide period.

The population attributable fraction (PAF) was calculated for men and women respectively to estimate the proportion of mental disorders that were associated with past year IPV. The population attributable fraction of a given mental disorder is thus the proportion of mental disorder in the total study population which could be avoided if no physical, sexual, psychological violence respectively were at hand. It was calculated using a modified formula to account for potential confounding [28,29].

The following formula was used for calculating the PAF: PAF = P_e_ (AOR-1)/(AOR)

Where:

P_e_ = proportion with mental disorders who were physically, sexually or psychologically abused.

AOR = Adjusted odds ratio for the association between IPV and a mental disorder of interest.

### Ethical considerations

The research protocol and tools were approved for scientific and ethical integrity by the Rwanda National Ethics Committee (Review Approval Notice N^o^ 165/RNEC/2011) and the National Institute of Statistics of Rwanda (N^o^ 1043/2011/10/NISR). The study strictly followed WHO guidelines on ethical issues related to violence-research [30], all participants were informed about their free choice to participate and to withdraw at whatever time they wanted during the study. Interviewers secured written consent from all respondents before the interview. To maintain confidentiality, the interview was conducted in privacy and with one interview in each household. Respondents were informed that questions could be sensitive and were reassured regarding the confidentiality of their responses. As IPV and mental disorders are delicate issues, it was agreed that those in need of any kind of assistance should be taken to a nearby health centre accompanied by the community health worker.

## Results

### Socio-demographic and psychosocial factors

A total of 440 (48%) men and 477 (52%) women, aged 20 to 35 years were enrolled in the study. The socio-demographic and psychosocial factors in this study reflected the life circumstances in Rwanda, where more than 80% of the population live in rural areas and approximatively 20% in urban areas. Only a small proportion of both men and women had completed primary level education (the majority being men) and only a small proportion had a higher income per month, the majority being men (Table 1). Further, a substantial proportion of both men and women had none of the household assets investigated, and hereby were considered extremely poor (26.6% and 30.6% respectively). Moreover, most of their partners had almost the same characteristics; a small number had completed primary school or vocational training. The majority of our study population were subsistence farmers, with small incomes and living in rather poor households.

**Table 1:** **Socio-demographic and psychosocial factors for men and women**

	**Total**		**Men**		**Women**		
	**(N = 917)**		**(n = 440)**		**(n = 477)**		
**Respondents characteristics**	***N***	**%**	***n***	**%**	***n***	**%**	p-value*
***1. Age groups in years***							
20-24	275	30.3	148	33.8	127	27.0	.050
25-29	300	33.0	144	32.9	156	33.2	
30-35	333	36.7	146	33.3	187	39.8	
***2. Marital status***							
Married or cohabiting	578	63.4	236	53.8	342	72.3	.000
Divorced or widowed	35	3.8	2	0.5	33	7.0	
Single	299	32.8	201	45.8	98	20.7	
***3. Level of education***							
Secondary school or university	117	15.2	50	13.3	67	17.0	.006
Complete primary school or vocational training	178	23.2	105	28.0	73	18.6	
Incomplete primary school	473	61.6	220	58.7	253	64.4	
***4. Personal income per month***							
More than 35,000Rwf	30	3.3	19	4.3	11	2.3	.005
17,500-35,000Rwf	55	6.0	36	8.2	19	4.0	
Less than 17,500Rwf	827	90.7	382	87.4	445	93.7	
***5. Social support***							
Good	140	15.3	77	17.5	63	13.2	.081
Poor	777	84.7	363	82.5	414	86.8	
**Partners characteristics**							
***6. Partner's age in years***							
20-24	97	16.8	67	28.2	30	8.8	.000
25-29	170	29.5	87	36.6	83	24.5	
30-35	310	53.7	84	35.3	226	66.7	
***7. Partners’ education level***							
Secondary school or university level	55	11.6	21	10.0	34	12.8	.412
Complete primary school or vocational level	141	29.7	59	28.1	82	30.9	
Incomplete primary school	279	58.7	130	61.9	149	56.2	
***8. Assets in the household***							
Improved (at least one of 8 assets)	654	71.3	323	73.4	331	69.4	.189
Poor (none of the 8 assets)	263	28.7	117	26.6	146	30.6	
**Respondents exposure to intimate partner violence**							
***9. Physical violence exposure***							
No	742	88.5	404	95.7	338	81.2	.000
Yes	96	11.5	18	4.3	78	18.8	
***10. Sexual violence exposure***							
No	742	90.6	404	98.5	338	82.6	.000
Yes	77	9.4	6	1.5	71	17.4	
***11. Psychological violence exposure***							
No	742	85.7	404	92.7	338	78.6	.000
Yes	124	14.3	32	7.3	92	21.4	

*p-value signifies statistical difference between men and women.

### Physical, sexual and psychological intimate partner violence

Of the participating women, 18.8% (n = 78) had been exposed to physical violence, 17.4% (n = 71) to sexual violence and 21.4% (n = 92) to psychological violence in the past 12 months (Table 1). Compared to women, few men reported intimate partner violence, only 4.3% (n = 18) were victims of physical violence, 1.5% (n = 6) had experienced sexual violence and 7.3% (n = 32) were victims of psychological violence in the past 12 months. Consequently, there was a statistically significant difference in prevalence for all types of IPV exposure between men and women (Table 1).

### Mental disorders

Women and men suffered from generalized anxiety disorder to about the same extent. Depressive disorders in the past two weeks and in earlier periods were more than two times more common in women than in men (women 26.5%, men 12.1%, and women 23.3%, men 8.4%, respectively). Similarly, suicide risk and PTSD cases were more than two times more common in women than in men (women 21.8%, men 9.6% and women: 19.6% and 7.1% respectively) (Table 2).

**Table 2:** **Prevalence of mental disorders for men and women**

	**Total population**		**Men**		**Women**		
**Mental disorders**	**N = 917**		**n = 440**		**n = 477**		
	***n***	**%**	***n***	**%**	***n***	**%**	p-value*
***1. Major depressive episodes in the past two weeks***							
No	736	80.4	386	87.9	350	73.5	.000
Yes	179	19.6	53	12.1	126	26.5	
***2. Major depressive episodes in earlier periods, of two weeks or more***							
No	764	83.9	401	91.6	363	76.7	.000
Yes	147	16.1	37	8.4	110	23.3	
***3. Suicide risk***							
No	769	84.0	396	90.4	373	78.2	.000
Yes	146	16.0	42	9.6	104	21.8	
***4. Post-Traumatic Stress Disorder (PTSD)***							
No	789	86.4	407	92.9	382	80.4	.000
Yes	124	13.6	31	7.1	93	19.6	
***5. Generalized anxiety Disorder***							
No	580	63.5	292	66.5	288	60.8	.074
Yes	333	36.5	147	33.5	186	39.2	

*p-value signifies statistical difference between men and women.

### Associations between IPV and mental disorders in women

Bivariate associations revealed that, for women, exposure to physical, sexual and psychological IPV considerably increased the risk of all the mental disorders explored in this study. Physical violence as well as psychological abuse were highly associated with major depressive episode in the past two weeks (OR 4.67, 2.77-7.87 and OR 4.84; 2.96-7.92 respectively), while sexual abuse were highly associated with generalised anxiety disorder (OR 5.31; 3.04-9.30) (Table 3).

**Table 3:** **Associations between physical, sexual and psychological violence and mental disorders in women (N = 477)**

**Variables**	**Major depressive episode in the past two weeks**			**Major depressive episode in earlier periods**			**Generalized anxiety disorder**			**Suicide risk**			**Post-traumatic stress disorder**		
		**Crude OR**	**Adjusted OR**		**Crude OR**	**Adjusted OR**		**Crude OR**	**Adjusted OR**		**Crude OR**	**Adjusted OR**	**n (%)**	**Crude OR**	**Adjusted OR**
	**n**	**OR**	**OR**	**n**	**OR**	**OR**	**n**	**OR**	**OR**	**n**	**OR**	**OR**	**n**	**OR**	**OR**
	**(%)**	**(95% CI)**	**(95% CI)**	**(%)**	**(95% CI)**	**(95% CI)**	**(%)**	**(95% CI)**	**(95% CI)**	**(%)**	**(95% CI)**	**(95% CI)**	**(%)**	**(95% CI)**	**(95% CI)**
***Physical violence***	40 (51.3)	4.67 (2.77-7.87)	4.63 (2.57-8.32)	33 (42.9)	3.44 (2.02-5.85)	2.88 (1.59-5.23)	51 (66.2)	4.38 (2.59-7.40)	4.70 (2.65-8.35)	33 (42.3)	3.62 (2.12-6.15)	4.54 (2.49-8.27)	26 (33.3)	2.79 (1.60-4.88)	3.16 (1.67-5.95)
Partners’ education			2.47 (1.33-4.55)			2.94 (1.55-5.56)			1.79 (1.07-2.98)			1.06 (0.59-1.92)			1.27 (0.68-2.38)
Assets in the household			2.33 (1.31-4.13)			2.25 (1.27-4.00)			1.25 (0.74-2.14)			0.96 (0.52-1.79)			0.98 (0.51-1.89)
Traumatic experience during the genocide			1.56 (0.88-2.75)			1.40 (0.79-2.48)			1.72 (1.04-2.83)			1.68 (0.95-2.98)			2.02 (1.10-3.70)
***Sexual violence***	37 (52.1)	4.83 (2.81-8.29)	5.49 (2.94-10.25)	27 (38.6)	2.88 (1.65-5.02)	2.74 (1.45-5.15)	50 (70.4)	5.31 (3.04-9.30)	6.37 (3.45-11.79)	26 (36.6)	2.85 (1.63-4.99)	3.73 (2.00-6.95)	25 (35.2)	3.04 (1.72-5.38)	4.20 (2.22-7.95)
Partners’ education			2.15 (1.15–4.02)			2.51 (1.32–4.77)			1.50 (0.89-2.54)			1.06 (0.58-1.96)			0.92 (0.49-1.73)
Assets in the household			2.66 (1.47-4.84)			2.46 (1.36-4.45)			1.45(0.83-2.52)			1.55 (0.83-2.92)			1.11 (0.57-2.17)
Traumatic experience during the genocide			1.92 1.06-3.47)			1.61 0.89-2.91)			1.91 (1.14-3.20)			1.80 (0.99-3.26)			1.86 (1.01-3.45)
***Psychological violence***	48 (52.2)	4.84 (2.96-7.92)	5.59 (3.19–9.80)	37 (40.7)	3.14 (1.90-5.19)	2.95 (1.66-5.21)	57 (62.6)	3.74 (2.31-6.07)	4.34 (2.54-7.43)	39 (42.4)	3.63 (2.20-5.99)	4.22 (2.37-7.49)	29 (31.5)	2.57 (1.51-4.38)	2.97 (1.62- 5.45)
Partners’ education			1.80 (1.00–3.24)			2.61 (1.40-4.88)			1.54 (0.93-2.55)			1.06 (0.59-1.91)			1.11 (0.59-2.06)
Assets in the household			2.44 (1.39-4.28)			2.53 (1.45-4.44)			1.30 (0.77-2.20)			1.35 (0.74-2.46)			1.44 (0.77-2.69)
Traumatic experience during the genocide			1.34 (0.77-2.32)			1.43 (0.82-2.50)			1.57 (0.96-2.56)			1.96 (1.12-3.42)			1.87 (1.04-3.38)

For women exposed to physical violence, the multivariate analyses showed that there was no change in odds ratios for depression in the past two weeks, for depression in earlier periods or for generalized anxiety disorder after controlling for socio-demographic and psychosocial variables (Table 3). Thus, the risk of depression in the past two weeks for women exposed to physical violence was more than four times higher (OR 4.63, 2.57-8.32) compared to unexposed women while exposure to sexual and psychological abuse gave adjusted odds ratios of 5.49 (2.94-10.25) and 5.59 (3.19-9.80) respectively compared to women unexposed to IPV. Additionally, physical, sexual and psychological violence remained statistically significant risk factors for suicide risk and PTSD, exhibiting slightly elevated odds ratios in the final models (Table 3).

Of interest is also to note that of the socio-demographic factors investigated in the multivariate analyses, partner’s low education and lack of assets in the household (as a proxy of socio-economic status) contributed to the risk of current and earlier depression. Similarly, partner’s education remained as a statistically significant risk factor for generalized anxiety disorder. Traumatic episodes that occurred 17 years earlier contributed significantly to the risk of PTSD, generalized anxiety disorder and suicide risk.

### Associations between IPV and mental disorders for men

The bivariate analyses showed that past year physical and psychological violence were highly associated with current major depressive episodes (OR 5.47, 2.01-14.87), earlier major depressive episodes (OR 3.54, 1.10-11.44) and generalized anxiety disorder for men (Table 4). After controlling for lack of assets in the household and exposure to traumatic episodes during the genocide period, past year physical and psychological violence remained a statistically significant risk factor for depression in the past two weeks (OR 4.17, 1.46-11.91 and 3.53, 1.54-8.08). Physical and psychological violence exposure also constituted statistically significant risk factors of magnitude for generalized anxiety disorder (OR 6.58, 2.08-20.81 and 4.19, 1.90-9.26) but not for earlier depressive episode when controlling for the same variables.

**Table 4:** **Associations between physical and psychological violence and mental disorders for men (N = 440)**

**Variables**	**Major depressive episode in the past 2 weeks**			**Major depressive episode in earlier periods**			**Generalized anxiety disorder**			**Suicide risk**			**Post-traumatic stress disorder**		
		**Crude OR**	**Adjusted OR**		**Crude OR**	**Adjusted OR**		**Crude OR**	**Adjusted OR**		**Crude OR**	**Adjusted OR**		**Crude OR**	**Adjusted OR**
	**n**	**OR**	**OR**	**n**	**OR**	**OR**	**n**	**OR**	**OR**	**n**	**OR**	**OR**	**n**	**OR**	**OR**
	**(%)**	**(95% CI)**	**(95% CI)**	**(%)**	**(95% CI)**	**(95% CI)**	**(%)**	**(95% CI)**	**(95% CI)**	**(%)**	**(95% CI)**	**(95% CI)**	**(%)**	**(95% CI)**	**(95% CI)**
***Physical violence***	7 (38.9)	5.47 (2.01-14.87)	4.17 (1.46-11.91)	4 (22.2)	3.54 (1.10-11.44)	2.09 (0.60-7.30)	14 (77.8)	7.88 (2.54-24.41)	6.58 (2.08–20.81)	3 (16.7)	1.97 (0.55-7.13)	1.44 (0.38-5.40)	2 (11.1)	1.74 (0.38-7.95)	1.45 (0.31-6.81)
Assets in the household			2.23 (1.19–4.20)			2.60 (1.24–5.44)			1.64 (1.03–2.61)			1.73 (0.87-3.44)			1.25 (0.55-2.84)
Traumatic experience during the genocide			2.05 (1.10-3.83)			4.40 (2.01-9.63)			1.83 (1.19-2.81)			2.18 (1.12-4.25)			1.62 (0.75-3.49)
***Psychological violence***	11 (34.4)	4.50 (2.03-9.98)	3.53 (1.54-8.08)	7 (21.9)	3.47 (1.39-8.69)	2.36 (0.89-6.21)	22 (68.8)	4.99 (2.29-10.84)	4.19 (1.90–9.26)	5 (15.6)	1.83 (0.66-5.03)	1.39 (0.49-3.95)	4 (12.5)	1.98 (0.65-6.07)	1.83 (0.58-5.74)
Assets in the household			1.89 (1.03-3.49)			1.97 (0.96-4.03)			1.49 (0.95-2.35)			1.67 (0.85-3.28)			1.04 (0.46-2.36)
Traumatic experience during the genocide			1.98 (1.09-3.59)			3.62 (1.74-7.52)			1.80 (1.18-2.73)			2.10 (1.10-4.03)			1.36 (0.64-2.87)

Physical and psychological violence did not remain statistically significant risk factors for suicide risk or PTSD when the analyses were adjusted for assets in the household and traumatic episodes in the genocide period.

Similarly for men, lack of assets in the household, i.e. extreme poverty contributed to the risk of depression, current and earlier as well as generalized anxiety disorder while having experienced traumatic episodes during the genocide contributed significantly to the risk of all mental disorders investigated except PTSD (Table 4).

### Population attributable fraction

The population attributable fraction (PAF) was used to calculate the contribution of physical, sexual and psychological violence to mental disorders for women and men.

If IPV could be eliminated among exposed women and men, the prevalence of serious mental disorders would be considerably decreased. For instance, for women, if physical and sexual violence were eliminated, generalized anxiety disorder would be reduced by 52% and 59% respectively. Likewise, for men, if physical and psychological violence were eliminated, generalized anxiety disorders would be reduced by 55% and 44% respectively (Table 5).

**Table 5:** **Population attributable fractions calculated for violence exposure and mental disorders as outcome variables for women and men**

	**Proportion of the outcome attributable to the exposure for women, N = 477**					
		Major depressive episodes past two weeks	Major depressive episodes in earlier periods	Suicide risk	PTSD	Generalized anxiety disorder
**Exposure**	Physical violence	0.40	0.28	0.33	0.23	0.52
	Sexual violence	0.43	0.25	0.27	0.27	0.59
	Psychological violence	0.43	0.27	0.32	0.21	0.48
	**Proportion of the outcome attributable to the exposure for men, N = 440**					
		Major depressive episodes past two weeks	Major depressive episodes in earlier periods	Suicide risk	PTSD	Generalized anxiety disorder
	Physical violence	0.23	0.07	0.04	0.03	0.55
	Psychological violence	0.19	0.09	0.03	0.05	0.44

## Discussion

This study provides important findings on the prevalence of mental disorders among young Rwandan men and women and the considerable contribution made by all forms of IPV to mental disorders in both men and women. The risk of current major depressive episodes, generalized anxiety disorder and suicide risk was more than three times elevated in women exposed to physical, sexual or psychological partner abuse as compared to women not exposed to any form of partner abuse. Furthermore, the population attributable fractions of intimate partner violence to mental disorders were high. Although men were considerably less exposed than women to violence exercised by the partner, they anyway suffered the risk of current major depressive episodes and generalized anxiety disorder when exposed to either physical or psychological abuse. These results are consistent with previous findings [7,10,15].

Other factors that seriously affected men’s and women’s mental health were poverty (lack of assets in the household) and experience of traumatic episodes in the genocide period, even though 17 years have elapsed since then. Partner’s poor educational attainment also contributed to mental disorders in women.

### Mental disorders in women

The general high prevalence of mental disorders in young women in this study, in which about one quarter suffered from major depressive episodes, generalized anxiety disorder, suicide risk and about one fifth from PTSD, reflects the numerous stressors in women’s everyday life. Our finding that women are at risk of carrying the double burden of IPV and poverty, causing additional stress, depression and other mental disorders is similarly found in other studies [5,31]. From Rwanda, high rates of mental disorders including depression and PTSD are reported to be associated with traumatic episodes during the genocide [21,25], which was also found in this study. Other studies from the post conflict settings further indicate that exposure to war related violence has well established associations with mental disorders including depression and PTSD [32-34].

For women, although adjustments were made for factors evidenced to be associated with mental health and particularly related to the Rwandan genocide exposure, associations between IPV and mental disorders in our study were convincing and in this way corroborate with findings from other low and middle income countries [8,35-37]. A recent review and a number of studies have further shown that depression, PTSD, suicide and anxiety are the most prevalent mental disorders related to IPV [5,36,38,39]. However, even if suicide risk was not the most prevalent disorder in our study but detected in one woman out of five, it needs further attention, as in some cases it might result in a suicide. Strong associations between all forms of violence and suicidal thoughts have been found in other studies using the WHO questionnaire [10,40].

Despite that IPV had strong associations with all forms of mental disorders, the partner’s low educational attainment and poverty (few assets in the household) similarly contributed to major depressive episode in the past two weeks and earlier, while traumatic experience in the genocide period was primarily associated with PTSD and suicide risk.

### Mental disorders in men

Our findings indicate that men suffer from mental disorders but to a lower degree than women, and IPV exposure was also lower compared to women. Few previous studies have included men in exploring the health effects of intimate partner violence and these are mainly from high income countries [41-43]. One of these studies, found no evidence of an association between IPV exposure and suicide attempts in men [42]. Another study reports that associations were at hand between IPV exposure and mental disorders, mainly with depressive symptoms [44] as did our findings. In our study, other factors, such as exposure to traumatic episodes during the genocide period and poverty were important risk factors for most of mental disorders for men in this study.

### The contribution of physical, sexual and psychological violence to mental disorders

As the population attributable fraction calculations indicated, any form of IPV has serious influence particularly on women’s mental health. Any strategy to improve women’s empowerment would possibly reduce the prevalence of mental disorders and in a longer perspective, the prevalence of IPV. A microfinance programme (loans) combined with a gender training intervention has been successful in other settings [45].

### Methodological considerations

This study randomly selected its population in a representative sample of young men and women in the southern province of Rwanda. A validated diagnostic tool for mental disorders and a well acknowledged, validated instrument for intimate partner violence were used. Interviews were performed by clinical psychologists with a good experience of scientific interviewing. The strength of this study is also the possibility to include exposure to traumatic episodes during the genocide period as an independent risk factor as several articles in the field from different post conflict areas have illustrated its long term impact on mental health status in men and women. For women, we hereby were able to show that partner violence was a strong independent risk factor also when the traumatic episodes during the genocide period variable was added to the analyses.

The variable ‘assets in the household’ was used as a proxy for socio-economic status as incomes were extremely small and the majority of the households had no regular income. Such estimates similarly have been used in the demographic health surveys undertaken in 2005 and in 2010 in Rwanda. However, this was a cross-sectional study, which limits interpretation of findings in relation to the direction of the association between IPV and mental disorders. However, a review article based on longitudinal studies provides considerable evidence to support the theory that a history of intimate partner violence precedes poor mental health outcome [5]. Additionally, under-reporting of intimate partner violence and mental disorders may be at hand due to their delicate nature and, the fear of disclosing such experiences. We believe that our results are most probably not overestimated.

## Conclusion

A life free of violence is a basic human right that every woman, man and child deserves. However, IPV is still a major contributing factor to mental disorders, especially in women. Given the high population attributable fraction, the prevention of IPV should be a public health priority as its prevention would contribute substantially to a reduction in mental disorders. Prevention activities have to challenge present social norms that support male authority and control over women. Gender equality and women’s empowerment need to be strengthened. Access to paid employment for both men and women would bring families out of poverty, improve women’s status and independence and possibly also reduce violence and mental disorders in families.

## Abbreviations

IPV: Intimate partner violence
MINI: Mini International Neuropsychiatric Interview
PTSD: Post traumatic stress disorders
PAF: Population attributable fraction
NCD: Non-communicable diseases
RwMHBC project: The Rwanda Violence, Mental Health and Barriers to Care project
SPH: School of Public Health
RNEC: Rwanda National Ethic Committee
NISR: National Institute of Statistics of Rwanda
WHO: World Health Organization
